# DNA repair gene XRCC3 Thr241Met polymorphism and susceptibility to glioma: A case-control study

**DOI:** 10.3892/ol.2014.2192

**Published:** 2014-05-28

**Authors:** GAOFENG XU, MAODE WANG, WANFU XIE, XIAOBIN BAI

**Affiliations:** Department of Neurosurgery, The First Affiliated Hospital of The Medical School of Xi’an Jiaotong University, Xi’an, Shaanxi 710061, P.R. China

**Keywords:** X-ray repair cross-complementing groups 3, glioma, polymorphism

## Abstract

The DNA repair gene, X-ray repair cross-complementing group 3 (XRCC3) Thr241Met polymorphism may be associated with a susceptibility to glioma. The present study aimed to investigate the association between the XRCC3 Thr241Met polymorphism and the potential susceptibility to gliomas. A hospital-based case-control study was conducted, which included a total of 886 patients with glioma and 886 healthy control subjects. Peripheral blood samples were extracted and the polymerase chain reaction-restriction fragment length polymorphism method was performed to analyze the genotypes. The glioma patients had a significantly higher frequency of the XRCC3 241 MetMet genotype [odds ratio (OR) = 1.62; 95% confidence interval (CI): 1.09–2.41; P=0.02] compared with the control subjects. When stratified by the grade of the glioma, the patients with stage IV glioma (according to the World Health Organization classification) had a significantly higher frequency of the XRCC3 241 MetMet genotype (OR=1.61; 95% CI: 1.06–2.44; P=0.03). When stratified by the histology of the glioma, there was no significant difference in the distribution of each genotype. The findings of the present study indicate that the XRCC3 Thr241Met polymorphism is associated with a susceptibility to glioma.

## Introduction

Gliomas account for >70% of all types of brain tumor (1). The estimated five-year survival rate is 60 and 74% for biopsy, and watchful waiting and early resection in low-grade gliomas, respectively (2,3). Gliomas are an enigmatic and heterogeneous disease, the exact etiology of which remains unclear (2). Certain factors are found to affect an individual’s glioma risk, such as hereditary genetic disorders (4), obesity during adolescence (5), being tall and exposure to high doses of ionizing radiation (5–8). Certain genome-wide association studies (GWAS) have reported that single nucleotide polymorphisms (SNPs) are associated with glioma susceptibility (9,10). However, the additional factors that contribute to glioma susceptibility require further investigation.

The DNA repair gene, X-ray repair cross-complementing group 3 (XRCC3), is involved in the process of homologous recombination repair for DNA double-strand breaks in order to maintain the stability of the genome (11). As the most common functional SNP, the XRCC3 Thr241Met (rs861539) polymorphism is at codon 241 in exon 7 with a C to T transition (12). The XRCC3 Thr241Met polymorphism has been investigated in various types of cancer and the results are mixed (13–17).

Although the association between the XRCC3 Thr241Met polymorphism and glioma risk have been extensively investigated, the currently available results are inconclusive (18–24). Although two meta-analyses have been conducted to investigate this association, conflicting results were yielded (23,24). The present study aimed to investigate the association between the XRCC3 Thr241Met polymorphism and the susceptibility to glioma.

## Subjects and methods

### Study population

This hospital-based case-control study was conducted in Northwest China. A total of 886 glioma patients and 886 healthy control subjects were recruited from the Department of Neurosurgery of the First Affiliated Hospital of The Medical School of Xi’an Jiaotong University (Xi’an, China) between January 2008 and January 2013. These subjects were recruited from the same geographical region. Alcohol consumption habits were defined as never (never drinks, or less than once a year), past and current. Smoking habits were defined as never (smoked <100 cigarettes in their lifetime), past and current. Tumor type and stage were determined according to the World Health Organization (WHO) criteria (25). Control subjects were matched with the patient group regarding gender, age and duration in education.

Informed consent was obtained from all the participants in the present study and, according to the Declaration of Helsinki, this study was approved by the Institutional Review Board of Xi’an Jiaotong University.

### DNA extraction and genotyping

Genomic DNA was extracted from peripheral blood samples by QIAamp DNA blood mini kits (QIAGEN Inc., Valencia, CA, USA) according to the manufacturer’s instructions. The XRCC3 Thr241Met polymorphisms were determined via polymerase chain reaction (PCR)-restriction fragment length polymorphism assay (PerkinElmer, Inc., Foster City, CA, USA). The primers were designed as follows: Forward, 5′-GCTGTCTCGGGGCATGGCTC-3′; and reverse, 5′-ACGAGCTCAGGGGTGCAACC-3′ to amplify a 208-bp fragment (26). The PCR products were digested overnight with the appropriate restriction enzyme, *Nla*III (New England Biolabs, Beverly, MA, USA). The XRCC3 241 Met allele was cut into two fragments of 120 and 88 bp, whereas the XRCC3 241Thr allele remained uncut with a length of 208 bp. The digested PCR products were resolved on 3% agarose gel and stained with ethidium bromide for visualization under an ultraviolet light. For quality control, the genotyping analysis was performed blind with regard to the subjects. The selected PCR-amplified DNA samples were also examined by DNA sequencing to verify the genotyping results.

### Statistical analysis

The χ^2^ test was used to assess for any deviation of the genotype frequencies from Hardy-Weinberg equilibrium and to compare the genotype distributions among glioma patients and healthy control subjects. The crude odds ratios (OR) and adjusted ORs for gender and age with a 95% confidence interval (CI) were calculated by logistic regression analysis. P<0.05 was considered to indicate a statistically significant difference. SAS statistical software, version 9.1 (SAS Institute Inc., Cary, NC, USA) was used to perform the statistical analyses.

## Results

The clinical characteristics of the patients with glioma and the healthy control subjects are presented in Table I. There were no significant differences identified in the distribution of gender, age, duration in education, smoking, alcohol consumption and family history of cancer (Table I). Among the 886 glioma patients, 655 had astrocytomas, 143 had ependymomas, 53 had oligodendrogliomas and 35 had mixed gliomas. Of these cases, 44 patients had grade I gliomas, 326 had grade II gliomas, 221 had grade III gliomas and 295 had grade IV gliomas (according to the WHO criteria). Genotype and allele frequencies were in Hardy-Weinberg equilibrium in the two groups.

Patients with glioma had a significantly higher frequency of the XRCC3 241 MetMet genotype (OR=1.62; 95% CI: 1.09–2.41; P=0.02) compared with the control subjects (Table II). When stratified by the grade of glioma, patients with stage IV glioma had a significantly higher frequency of the XRCC3 241 MetMet genotype (OR=1.61; 95% CI: 1.06–2.44; P=0.03; Table III). When stratified by the histology of glioma, there was no significant difference identified in the distribution of each genotype (Table III).

## Discussion

The accumulating genetic evidence concerning the risk of glioma is strongly positive. Previous GWAS have reported that SNPs are associated with glioma susceptibility (9,10). A meta-analysis of 11 case-control studies with 2,404 glioma cases and 6,379 control subjects found that the risk of glioma significantly increased between the GSTP1 A114V genotype and other histopathological gliomas, not including glioblastoma multiforme (27). A meta-analysis of nine case-control studies with 3,146 cases and 4,296 control subjects indicated that the XRCC1 Arg399Gln polymorphism was associated with an increased risk of glioma among Asian individuals and borderline increased risk for glioblastoma among Caucasian individuals, whereas the XRCC1 Arg194Trp/Arg280His polymorphisms may have no affect on the susceptibility of glioma among different ethnicities (24). A systematic literature review and meta-analysis of six case-control studies with 2,362 glioma cases and 3,085 control subjects did not indicate a major role of the XRCC1 Arg399Gln polymorphism in influencing the risk of glioma among Caucasian individuals (28). A meta-analysis of 11 case-control studies with 3,810 cases and 6,079 control subjects reported that the XRCC1 Arg399Gln polymorphism was moderately associated with an increased risk of gliomas in Asian individuals, while the XRCC1 Arg194Trp and XRCC1 Arg280His polymorphisms demonstrated no significant affects (29). A meta-analysis of 11 case-control studies with 2,808 glioma cases and 3,114 control subjects identified that the XRCC1 Arg399Gln polymorphism may contribute to the susceptibility to gliomas in Asian individuals (30).

The XRCC3 Thr241Met polymorphism has been investigated in various types of cancer and the findings have been varied. A meta-analysis of 157 case-control studies reported the participation of XRCC3 T241M in the susceptibility for bladder and breast cancer, particularly in Caucasian individuals, and the XRCC3 T241M polymorphism was associated with a decreased risk of lung cancer (31). However, another meta-analysis of 17 case-control studies indicated that the XRCC3 T241M polymorphism was not associated with lung cancer risk, which was a stratified analysis by ethnicity, histology and smoking status (32). A meta-analysis of 15 case-control studies involving 4,475 cases and 6,373 control subjects hypothesized that the XRCC3 Thr241Met polymorphism may modify the risk of colorectal cancer, particularly in Asian individuals (33). Conversely, another meta-analysis of 23 published case-control studies indicated that the XRCC3 Thr241Met polymorphism was not associated with the risk of colorectal cancer (34). A meta-analysis of case-control studies identified that the XRCC3 241M allele may act as a head and neck cancer risk factor among all subjects (16). In addition, the XRCC3 241M allele may act as a risk factor for breast cancer (35).

The present study has various major limitations. First, these findings should be interpreted with caution as the study population was from Northwest China, which reduces the potential for confounding from ethnicity and does not permit extrapolation of the results to other ethnic groups. Additionally, since the control subjects were recruited from those individuals who were at hospitals for a routine health examination, there was a certain risk of selection bias. Finally, the interactions between gene-gene, gene-environment and even different polymorphic loci of the same gene may modulate the glioma risk.

In conclusion, the present study indicates that the XRCC3 Thr241Met polymorphism is associated with a susceptibility to glioma. However, further studies within Chinese populations with larger sample sizes are required.

## Figures and Tables

**Table I:** Characteristics of the glioma and healthy control subjects.

Characteristic	Glioma, n=886	Control, n=886	P-value
Gender, n (%)			
Male	487 (55.0)	483 (54.5)	0.85
Female	399 (45.0)	403 (45.5)	0.85
Mean age ± SD (years)	41.8±8.6	42.0±9.0	0.63
Mean time spent in education ± SD (years)^a^	11.5±2.5	11.6±2.6	0.41
Smoker, n (%)			
Never	488 (55.1)	480 (54.2)	0.84
Past	89 (10.0)	93 (10.5)	0.78
Current	309 (34.9)	313 (35.3)	0.89
Alcohol consumption, n (%)			
Never	530 (59.8)	542 (61.2)	0.77
Past	149 (16.8)	145 (16.4)	0.83
Current	207 (23.4)	199 (22.4)	0.72
Family history of cancer, n (%)			
No	797 (90.0)	795 (89.7)	0.88
Yes	89 (10.0)	91 (10.3)	0.88
Histology, n (%)			
Astrocytomas	655 (73.9)		
Ependymomas	143 (16.1)		
Oligodendrogliomas	53 (6.0)		
Mixed gliomas	35 (4.0)		
WHO grade, n (%)			
I	44 (5.0)		
II	326 (36.8)		
III	221 (24.9)		
IV	295 (33.3)		

a This parameter was included as the time spent in education has been found to collate with living habits.

WHO, World Health Organization SD, standard deviation.

**Table II:** Genotypes and allele distribution of X-ray repair cross-complementing group 3 Thr241Met polymorphism among the glioma and healthy control subjects.

Genotype	Glioma, n (%)	Control, n (%)	OR (95% CI)	P-value
ThrThr	472 (53.3)	485 (54.7)	1.00 (Reference)	
ThrMet	343 (38.7)	356 (40.2)	0.99 (0.82–1.20)	0.92
MetMet	71 (8.0)	45 (5.1)	1.62 (1.09–2.41)	0.02
Thr allele frequency	1,287 (72.6)	1,326 (74.8)	1.00 (Reference)	
Met allele frequency	485 (27.4)	446 (25.2)	1.12 (0.97–1.30)	0.14

OR, odds ratio; CI, confidence interval; Thr, threonine; Met, methionine.

**Table III:** Stratification analysis of X-ray repair cross-complementing group 3 Thr241Met polymorphism in glioma patients.

A, Histology										

		ThrThr			ThrMet			MetMet		
				
Type	Cases	n (%)	OR (95% CI)	P-value	n (%)	OR (95%CI)	P-value	n (%)	OR (95% CI)	P-value
Astrocytomas	655	348 (53.1)	1.00 (0.84–1.18)	0.98	255 (38.9)	1.01 (0.83–1.22)	0.95	52 (7.9)	0.99 (0.68–1.44)	0.96
Ependymomas	143	77 (53.8)	1.01 (0.75–1.36)	0.94	54 (37.8)	0.98 (0.70–1.37)	0.89	12 (8.4)	1.05 (0.55–1.98)	0.89
Oligodendrogliomas	53	29 (54.7)	1.03 (0.64–1.64)	0.91	20 (37.7)	0.98 (0.57–1.66)	0.93	4 (7.6)	0.94 (0.33–2.68)	0.91
Mixed gliomas	35	18 (51.4)	0.97 (0.54–1.72)	0.91	14 (40.0)	1.03 (0.55–1.94)	0.92	3 (8.6)	1.07 (0.32–3.56)	0.91
Total	886	472 (53.3)	1.00 (Reference)		343 (38.7)	1.00 (Reference)		71 (8.0)	1.00 (Reference)	

B, World Health Organization classification										

		ThrThr			ThrMet			MetMet		
				
Grade	Cases	n (%)	OR (95% CI)	P-value	n (%)	OR (95%CI)	P-value	n (%)	OR (95% CI)	P-value

I	44	23 (52.3)	0.98 (0.59–1.65)	0.94	18 (40.9)	1.06 (0.60–1.85)	0.85	3 (6.8)	0.85 (0.26–2.81)	0.79
II	326	185 (56.8)	1.07 (0.86–1.32)	0.56	125 (38.3)	0.99 (0.78–1.26)	0.94	16 (4.9)	0.61 (0.35–1.07)	0.09
III	221	124 (56.1)	1.05 (0.82–1.35)	0.68	83 (37.6)	0.97 (0.73–1.29)	0.83	14 (6.3)	0.79 (0.44–1.43)	0.44
IV	295	140 (47.4)	0.89 (0.71–1.12)	0.33	117 (39.7)	1.02 (0.80–1.31)	0.85	38 (12.9)	1.61 (1.06–2.44)	0.03
Total	886	472 (53.3)	1.00 (Reference)		343 (38.7)	1.00 (Reference)		71 (8.0)	1.00 (Reference)	

Thr, threonine; Met, methionine; OR, odds ratio; CI, confidence interval.

## Abbreviations

XRCC3: X-ray repair cross-complementing group 3
PCR: polymerase chain reaction
OR: odds ratio
CI: confidence interval
GWAS: genome-wide association studies
SNPs: single-nucleotide polymorphisms

## References

[1] Wen PY, Kesari S (2008). Malignant gliomas in adults. N Engl J Med.

[2] Ohgaki H, Kleihues P (2005). Epidemiology and etiology of gliomas. Acta Neuropathol.

[3] Jakola AS, Myrmel KS, Kloster R (2012). Comparison of a strategy favoring early surgical resection vs a strategy favoring watchful waiting in low-grade gliomas. JAMA.

[4] Reuss D, von Deimling A (2009). Hereditary tumor syndromes and gliomas. Recent Results Cancer Res.

[5] Moore SC, Rajaraman P, Dubrow R (2009). Height, body mass index, and physical activity in relation to glioma risk. Cancer Res.

[6] Kostron H, Swartz MR, Miller DC, Martuza RL (1986). The interaction of hematoporphyrin derivative, light, and ionizing radiation in a rat glioma model. Cancer.

[7] Hocking B (2008). Occupational exposure to ionizing and non-ionizing radiation and risk of glioma. Occup Med (Lond).

[8] Ron E, Modan B, Boice JD (1988). Tumors of the brain and nervous system after radiotherapy in childhood. N Engl J Med.

[9] Shete S, Hosking FJ, Robertson LB (2009). Genome-wide association study identifies five susceptibility loci for glioma. Nat Genet.

[10] Wrensch M, Jenkins RB, Chang JS (2009). Variants in the CDKN2B and RTEL1 regions are associated with high-grade glioma susceptibility. Nat Genet.

[11] Griffin CS, Simpson PJ, Wilson CR, Thacker J (2000). Mammalian recombination-repair genes XRCC2 and XRCC3 promote correct chromosome segregation. Nat Cell Biol.

[12] Shen MR, Jones IM, Mohrenweiser H (1998). Nonconservative amino acid substitution variants exist at polymorphic frequency in DNA repair genes in healthy humans. Cancer Res.

[13] Zhao Y, Deng X, Wang Z, Wang Q, Liu Y (2012). Genetic polymorphisms of DNA repair genes XRCC1 and XRCC3 and risk of colorectal cancer in Chinese population. Asian Pac J Cancer Prev.

[14] Cheng CX, Xue M, Li K, Li WS (2012). Predictive value of XRCC1 and XRCC3 gene polymorphisms for risk of ovarian cancer death after chemotherapy. Asian Pac J Cancer Prev.

[15] Shaker OG, Sadik NA (2012). Polymorphisms in interleukin-10 and interleukin-28B genes in Egyptian patients with chronic hepatitis C virus genotype 4 and their effect on the response to pegylated interferon/ribavirin-therapy. J Gastroenterol Hepatol.

[16] Yin QH, Liu C, Li L, Zu XY, Wang YJ (2012). Association between the XRCC3 T241M polymorphism and head and neck cancer susceptibility: a meta-analysis of case-control studies. Asian Pac J Cancer Prev.

[17] Zhu X, Zhong Z, Zhang X (2012). DNA repair gene XRCC3 T241M polymorphism and bladder cancer risk in a Chinese population. Genet Test Mol Biomarkers.

[18] Zhao P, Zou P, Zhao L (2013). Genetic polymorphisms of DNA double-strand break repair pathway genes and glioma susceptibility. BMC Cancer.

[19] Pan WR, Li G, Guan JH (2013). Polymorphisms in DNA repair genes and susceptibility to glioma in a chinese population. Int J Mol Sci.

[20] Luo KQ, Mu SQ, Wu ZX, Shi YN, Peng JC (2013). Polymorphisms in DNA repair genes and risk of glioma and meningioma. Asian Pac J Cancer Prev.

[21] Kiuru A, Lindholm C, Heinävaara S (2008). XRCC1 and XRCC3 variants and risk of glioma and meningioma. J Neurooncol.

[22] Wang LE, Bondy ML, Shen H (2004). Polymorphisms of DNA repair genes and risk of glioma. Cancer Res.

[23] Zhao B, Ye J, Li B, Ma Q, Su G, Han R (2013). DNA repair gene XRCC3 Thr241Met polymorphism and glioma risk: a meta-analysis. Int J Clin Exp Med.

[24] Jiang J, Quan XF, Zhang L, Wang YC (2013). The XRCC3 Thr241Met polymorphism influences glioma risk - a meta-analysis. Asian Pac J Cancer Prev.

[25] Louis DN, Ohgaki H, Wiestler OD, Cavenee WK, Burger PC (2007). The 2007 WHO classification of tumours of the central nervous system. Acta Neuropathol.

[26] Duan Z, Shen H, Lee JE (2002). DNA repair gene XRCC3 241Met variant is not associated with risk of cutaneous malignant melanoma. Cancer Epidemiol Biomarkers Prev.

[27] Fan Z, Wu Y, Shen J, Zhan R (2013). Glutathione S-transferase M1, T1, and P1 polymorphisms and risk of glioma: a meta-analysis. Mol Biol Rep.

[28] Jacobs DI, Bracken MB (2012). Association between XRCC1 polymorphism 399 G→A and glioma among Caucasians: a systematic review and meta-analysis. BMC Med Genet.

[29] Sun JY, Zhang CY, Zhang ZJ (2012). Association between XRCC1 gene polymorphisms and risk of glioma development: a meta-analysis. Asian Pac J Cancer Prev.

[30] Wei X, Chen D, Lv T (2013). A functional polymorphism in XRCC1 is associated with glioma risk: evidence from a meta-analysis. Mol Biol Rep.

[31] Fan W, Li S, Chen Q, Huang Z, Ma Q, Xiao Z (2013). Association between interleukin-10 promoter polymorphisms and endometriosis: a meta-analysis. Gene.

[32] Zhan P, Wang Q, Qian Q, Yu LK (2013). XRCC3 Thr241Met gene polymorphisms and lung cancer risk: a meta-analysis. J Exp Clin Cancer Res.

[33] Wang Z, Zhang W (2013). Association between XRCC3 Thr241Met polymorphism and colorectal cancer risk. Tumour Biol.

[34] Zhu H, Lei X, Liu Q, Wang Y (2013). Interleukin-10-1082A/G polymorphism and inflammatory bowel disease susceptibility: a meta-analysis based on 17,585 subjects. Cytokine.

[35] Li D, He Q, Li R, Xu X, Chen B, Xie A (2012). Interleukin-10 promoter polymorphisms in Chinese patients with Parkinson’s disease. Neurosci Lett.

